# The Healthy Human Blood Microbiome: Fact or Fiction?

**DOI:** 10.3389/fcimb.2019.00148

**Published:** 2019-05-08

**Authors:** Diego J. Castillo, Riaan F. Rifkin, Don A. Cowan, Marnie Potgieter

**Affiliations:** ^1^Department of Biochemistry, Genetics and Microbiology, Centre for Microbial Ecology and Genomics, University of Pretoria, Pretoria, South Africa; ^2^Human Origins and Palaeo Environmental Research Group, Department of Anthropology and Geography, Oxford Brookes University, Oxford, United Kingdom

**Keywords:** human blood microbiome, bacteria, dysbiosis, disease, contamination

## Abstract

The blood that flows perpetually through our veins and arteries performs numerous functions essential to our survival. Besides distributing oxygen, this vast circulatory system facilitates nutrient transport, deters infection and dispenses heat throughout our bodies. Since human blood has traditionally been considered to be an entirely sterile environment, comprising only blood-cells, platelets and plasma, the detection of microbes in blood was consistently interpreted as an indication of infection. However, although a contentious concept, evidence for the existence of a healthy human blood-microbiome is steadily accumulating. While the origins, identities and functions of these unanticipated micro-organisms remain to be elucidated, information on blood-borne microbial phylogeny is gradually increasing. Given recent advances in microbial-hematology, we review current literature concerning the composition and origin of the human blood-microbiome, focusing on bacteria and their role in the configuration of both the diseased and healthy human blood-microbiomes. Specifically, we explore the ways in which dysbiosis in the supposedly innocuous blood-borne bacterial microbiome may stimulate pathogenesis. In addition to exploring the relationship between blood-borne bacteria and the development of complex disorders, we also address the matter of contamination, citing the influence of contaminants on the interpretation of blood-derived microbial datasets and urging the routine analysis of laboratory controls to ascertain the taxonomic and metabolic characteristics of environmentally-derived contaminant-taxa.

## Introduction

The human microbiome comprises a vast corpus of bacterial, archaeal, viral and fungal microbial taxa. While most of these micro-organisms are commensal, many are mutualistic and some are pathogenic. Regardless of whether their presence is beneficial, inconsequential or detrimental, our lives are inextricably linked to the microbes with which we share our bodies. In fact, despite being 1,000 times smaller than human cells, bacteria comprise ~2% of the adult human body mass (1.5 kg), roughly equivalent in size to the human brain or liver (Molina and DiMaio, 2012). Given our extensive co-evolutionary history with microbes (Moeller et al., 2016), it is not surprising that the estimated number of unique bacterial genes in our “accessory genome” (~3,300,000) exceeds the number of our own genes (~22,000) by a factor of 150 (Qin et al., 2010). Human microbiome research, described as the study of the entire DNA content of micro-organisms inhabiting our bodies, has rapidly evolved over the past decade. As this topic has been reviewed extensively elsewhere (Cho and Blaser, 2012; Morgan et al., 2012; Kim et al., 2013; Khanna and Tosh, 2014; Lloyd-Price et al., 2016), we focus here on current evidence indicative of the existence of a “healthy” human blood-microbiome (HBM).

The exploration of our “microbial-selves” has been facilitated largely by the introduction of Next Generation Sequencing (NGS) and the advent of whole metagenome shotgun sequencing (WMGS) as techniques to study microbial genetic material present in different human body-sites (Segata et al., 2013). For many years, scientists have aimed to establish a taxonomy-based set of core human-associated micro-organisms. However, a more valuable approach involves ascertaining the primary core microbial composition based on functional (metabolic) capacity, since it is easier to correlate pathogenesis with deviations or changes (i.e., dysbiosis) in a “core” microbiome (Turnbaugh et al., 2009). In this regard, several large-scale population-based studies have sequenced the metagenomes of the human intestinal-microbiome (IM), as well as other medically-relevant body-sites including the skin, vagina and mouth. Two notable collaborative projects have been developed to achieve this fundamental aim. As part of the “Metagenomes of the Human Intestinal Tract” project (Qin et al., 2010; Le Chatelier et al., 2013; Li et al., 2014) and the “Human Microbiome Project” (HMP) (Aagaard et al., 2013), more than 2,000 people from across the globe had contributed to the study of the microbiome structure of healthy individuals since 2006 (Lloyd-Price et al., 2016). Although most contemporary research focuses on the human IM, the microbial communities present in the human mouth and eyes, on the skin, lungs and in the placenta and urogenital tracts have also been described (Aagaard et al., 2013; Blekhman et al., 2015; Lloyd-Price et al., 2016).

**Figure 1:**
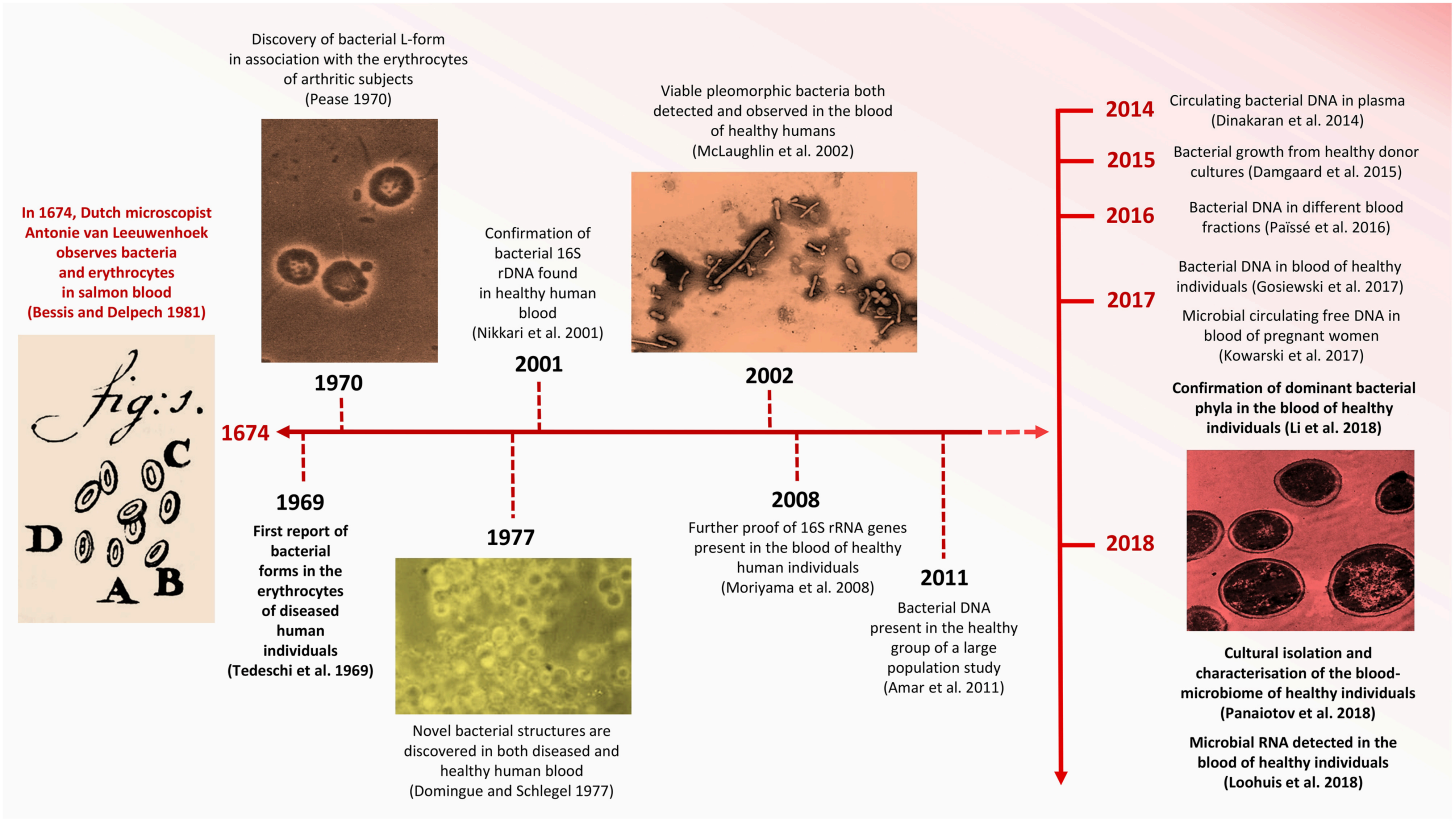
Timeline indicating significant advances concerning HBM research. Images modified from Pease (1970), Domingue and Schlegel (1977), Bessis and Delpech (1981), McLaughlin et al. (2002), and Panaiotov et al. (2018).

More recently, the prospect of the existence of a “healthy” HBM has roused much interest in the scientific community (McLaughlin et al., 2002; Bahrani-Mougeot et al., 2008; Païssé et al., 2016). Human blood comprises ~54.3% plasma, ~45% red blood cells (erythrocytes), ~0.7% white blood cells (lymphocytes) and a variable number of platelets (thrombocytes), depending on health status (Alberts et al., 2002). Following the first documented observation of erythrocytes by Antonie van Leeuwenhoek in 1674 (Bessis and Delpech, 1981) (Figure 1), it is now known that blood is the liquid medium that carries and sustains the most basic, but most essential, elements of life. Whereas erythrocytes are responsible primarily for the transport of oxygen, lymphocytes serve as a highly efficient surveillance system that monitors the blood for invasive microbes (Jerne, 1973). The primary function of thrombocytes is to react to bleeding from blood vessel injury by clotting (Blache, 1992). Because blood has traditionally been considered to be a sterile environment, devoid of all other forms of foreign (e.g., bacterial) cells, it is not surprising that the concept of a healthy HBM has been met with criticism (Nikkari et al., 2001; McLaughlin et al., 2002; Païssé et al., 2016). While evidence for the existence of a blood-microbiome in various domesticated mammals and birds do exist (Sze et al., 2014; Mandal et al., 2016; Vientós-Plotts et al., 2017), we focus on the healthy human blood-microbiome. Given that an increasing number of studies are exploring the notion that the presence of “foreign” micro-organisms in human blood does not necessarily equate with infection or with a diseased state, we review evidence concerning the discovery and tentative acceptance of the healthy HBM. In addition, we explore the potential origins and identities of “resident” micro-organisms, their phylogenetic affiliations and the clinical relevance of an allegedly healthy HBM. We also address the adverse influence that contaminants derived from reagents and laboratory environments exert on sequence-based IM and HBM research and the recovery of numerous microbial taxa in DNA extraction and library preparation controls.

## Controversy and Evolution of a Novel Concept

The controversy concerning the incidence of foreign cells in human blood extends back to the late 1960s, when Tedeschi et al. (1969) reported the presence of metabolically active bacteria in the blood of healthy human subjects (Figure 1). Specifically, the increased absorption of nucleosides and amino acids in erythrocyte suspensions led them to hypothesize that mycoplasm-like or L-phase (cell-wall deficient) bacteria were present in the blood of overtly non-diseased individuals. Nearly a decade later, in 1977, Gerald Domingue and Jorgen Schlegel reported that ~7% of blood samples derived from a healthy human cohort exhibited bacterial growth following osmotic lysis and filtering (Domingue and Schlegel, 1977).

More recent evidence for the hypothetical existence of a healthy HBM derives from Nikkari et al. (2001) who reported the presence of bacterial DNA in the blood of a healthy human cohort. This study, based on qPCR, included the use of rRNA-specific fluorescent probes and 16S rRNA gene-specific primers and identified bacterial taxa belonging to five divisions and seven phylogenetic groups. This study is, however, limited by the fact that all observations were based on the analysis of the blood of only four individuals (Table 1). Shortly thereafter, McLaughlin et al. (2002) described the presence of pleomorphic bacteria in the blood of individuals who did not present any perceptible clinical manifestations of disease. In this study, transmission electron microscopy (TEM), dark-field microscopy (DFM), fluorescence *in situ* hybridization (FISH) and the sequencing of PCR-amplified 16S rRNA and *gyrB* genes confirmed the presence of bacterial DNA in the blood of healthy individuals. Correspondingly, Moriyama et al. (2008) contributed to the concept of a “healthy” HBM by confirming the presence of bacterial 16S rRNA genes in the blood of healthy humans.

**Table 1:** HBM studies concerning both healthy and diseased human participants.

**Study population**	**Healthy HBM indications**	**Method of detection**	**Major findings**	**Shortcomings**	**References**
One-hundred male and female subjects selected at random.	Cohort consisted of healthy individuals only.	Radioactive uptake of nucleosides and amino acids in erythrocyte suspensions.	Possible presence of mycoplasm-like or L-phase bacterial forms in the bloodstream of overtly healthy individuals.	Fails to report the use of controls. Outdated technology.	Tedeschi et al., 1969
Ninety-five diseased patients and 60 healthy individuals.	Bacterial growth observed in 7% of the healthy cohort.	Filtrated blood used to culture bacteria.	Novel bacterial structures seen in blood suggesting bacterial phase before becoming ordinary bacteria.	No biochemical or genetic data in support of morphological observations.	Domingue and Schlegel, 1977
Four individuals without clinical signs of disease.	Study population consisted of healthy individuals only.	qPCR including the use of rRNA gene-specific fluorescent probes.	Presence of bacteria from five divisions and 7 distinct phylogenetic groups in the blood of healthy individuals.	Very small (*n* = 4) sample size.	Nikkari et al., 2001
Twenty-five healthy individuals.	Study population consisted of healthy individuals only.	PCR amplification of 16S rRNA and *gyrB* genes. Used microscopy (dark-field and TEM) and fluorescent *in situ* hybridization (FISH).	Confirms the existence of pleomorphic bacteria in “healthy” blood. Bacteria exhibited limited growth and susceptibility to antibiotics. Sequencing revealed the presence of *Proteobacteria* (possibly *Pseudomonas*).	Pleomorphic structures could be RBC-derived microparticles.	McLaughlin et al., 2002
Two healthy individuals.	Study population consisted of healthy individuals only.	PCR of 16S rRNA gene and Sanger sequencing of different clones.	16S rRNA genes in “healthy” blood confirmed. Bacteria identified only in clones (*Aquabacterium, Budvicia, Stenotrophomonas, Serratia, Bacillus* and *Flavobacteria*).	Very small (*n* = 2) sample size.	Moriyama et al., 2008
Comprising 3,280 patients from the DESIR study.	Healthy (non-diabetic) patients also presented bacterial DNA in their blood.	Quantification of 16S rRNA gene with broad-range quantification kit and pyro-sequencing.	16S rRNA gene concentration higher in individuals that developed diabetes. A core blood microbiome, mostly consisting of *Proteobacteria*, found in all study groups.	Conclusions solely based on the finding of bacterial DNA.	Amar et al., 2011
Comprising 3,936 patients from the DESIR study.	Bacterial DNA in blood of individuals not presenting CVD.	16S rRNA gene qPCR.	*Eubacteria* and *Proteobacteria* identified in groups affected by and free of cardiovascular disease.	Conclusions solely based on the finding of bacterial DNA.	Amar et al., 2013
Eighty CVD patients and 40 healthy individuals.	Healthy control group presented circulating bacterial DNA in their plasma.	16S rRNA and β globin qPCR and shotgun sequencing of circulating DNA from blood plasma.	Bacterial DNA and microbial diversity higher in CVD group. *Proteobacteria* and *Actinobacteria* dominant in CVD group (*Actinobacteria* and *Proteobacteria* dominant in control group).	Fails to report the use of positive and negative controls.	Dinakaran et al., 2014
Fifty type-2 diabetes patients and 50 control individuals.	Bacterial rRNA detected in 4% of healthy individuals (opposed to 28% in diabetes patients).	16S rRNA RT-qPCR.	Higher detection rate of potential IM bacteria in the blood of patients with type-2 diabetes than in control group.	Fails to report the use of negative and positive controls.	Sato et al., 2014
Sixty self-reported healthy individuals older than 49 years.	Bacterial growth observed in 62% of healthy individuals.	Blood suspensions incubated on trypticase soy blood agar (TSA) or blue lactose plates, and identified by 16S rRNA gene colony PCR.	Bacterial growth observed in 35% of RBC fractions and 53% of plasma fractions. *Staphylococci, Propionibacterium, Micrococcus* and *Bacillus* most frequently found.	Method unable to detect unculturable bacteria.	Damgaard et al., 2015
Thirty healthy blood donors (18 to 53 years old).	Study population consisted of healthy individuals only.	16S rRNA gene qPCR and 16S targeted metagenomic sequencing (Illumina MiSeq).	Bacterial DNA present in buffy coat, erythrocytes and plasma. Most bacterial DNA corresponds to *Proteobacteria* and *Actinobacteria (Firmicutes* and *Bacteroidetes* also found).	Conclusions solely based on the finiding of bacterial DNA.	Païssé et al., 2016
Twenty-three healthy individuals and 62 patients with sepsis.	Bacterial DNA found in all samples including 23 healthy individuals.	16S rRNA gene targeted metagenomic NGS (Illumina MiSeq).	Healthy samples presented higher diversity than sepsis patients. Abundance of *Proteobacteria* decreased in healthy samples, while *Actinobacteria* decreased in sepsis patients.	Conclusions solely based on the finiding of bacterial DNA.	Gosiewski et al., 2016
Nine cirrhosis patients and 9 healthy individuals (~60 years of age).	Bacteria found in 2 out of the 9 control individuals.	Microbial DNA qPCR (16S rRNA target gene).	Number of bacterial species and amount of bacterial DNA increased in cirrhotic patients.	Very small sample size (*n* = 9).	Traykova et al., 2017
Twenty-one bone marrow transplant-, 59 lung transplant- and 76 heart-transplant patients (32 pregnant participants).	Sequencing reads obtained from all transplant patients and pregnant participants.	Shotgun sequencing of cell-free DNA. Confirmation of novel contigs by direct PCR.	Circulating free-DNA from novel uncharacterized bacteria and viruses that could be members of the human IM found in the blood of all participants.	Conclusions solely based on the finiding of bacterial DNA.	Kowarsky et al., 2017
Fifty patients with severe acute pancreatitis and 12 healthy individuals.	Bacterial DNA found in all healthy participants.	16S rDNA gene qPCR and targeted metagenomic sequencing using Ion Torrent.	Higher number of 16S rDNA gene copies in patients. Healthy phyla include *Proteobacteria, Actinobacteria, Firmicutes* and *Bacteroidetes*. Increase of *Bacteroidetes* and a decrease of *Actinobacteria* observed in patients.	Small samples size (*n* = 12) for healthy cohort. Conclusions based solely on the detection of bacterial DNA.	Li et al., 2018
Twenty-eight blood samples from healthy individuals.	All blood samples were culture positive (confirmed by gram staining and TEM).	16S rRNA genes and ITS2 targeted sequencing on Illumina MiSeq and TEM.	Cultural isolation and characterization of the blood- microbiome of healthy individuals.	Fails to report the use of negative and positive controls.	Panaiotov et al., 2018
Comprising 192 individuals (48 with schizophrenia, 47 with lateral sclerosis, 48 with bipolar disorder and 49 healthy).	Bacterial transcripts identified in blood samples from healthy individuals.	High throughput RNA sequencing.	Prevalent phyla across study groups were *Proteobacteria, Firmicutes* and *Cyanobacteria*. Microbial diversity in schizophrenia patients significantly increased.	RNA analyses does not confirm all blood-borne icroorganisms.	Loohuis et al., 2018
Ten participants: five healthy subjects and five suffering from asthma.All women	Bacterial DNA and RNA in blood of all healthy individuals.	16S rRNA gene sequencing. *De novo* assembly of unmapped mRNA reads, and culturing.	From DNA and RNA analysis, most abundant phyla were *Proteobacteria, Actinobacteri*a, *Firmicutes* and *Bacteroidetes*. Blood microbiome more similar to skin and oral communities.	Small sample size (*n* = 5 per group).	Whittle et al., 2018
Fifty patients suffering from diabetes type two, and 100 healthy control individuals	Bacterial DNA found in all healthy participants.	16S rRNA genes targeted sequencing on Illumina MiSeq.	Participants that carried the genus *Sediminibacterium* had an increased risk of diabetes. Those with *Bacteroides* genus presented a lower risk of developing the disease.	Conclusions solely based on the finiding of bacterial DNA.	Qiu et al., 2019

*We emphasize the detection of bacteria in the blood of healthy human cohorts*.

As anticipated, challenging the traditional conviction concerning the sterility of blood in healthy humans under normal circumstances has generated considerable controversy. Mitchell et al. (2016) assessed the findings of McLaughlin et al. (2002) and other studies, concluding that the pleomorphic bacteria identified in the blood of healthy humans were, in fact, nothing more than micro-particles derived from disintegrated erythrocytes. Martel et al. (2017) supported this argument with the discovery that bacteria-like structures closely resembled membrane vesicles and that vibrating refringent particles captured by dark-field microscopy were merely aggregates of blood proteins. Although the visual confirmation of micro-organisms present in the blood of healthy individuals requires further examination, evidence confirming the presence of microbial genetic material in the blood-circulatory system is accumulating (McLaughlin et al., 2002; Moriyama et al., 2008; Païssé et al., 2016).

The application of innovative analytical technologies, such as targeted NGS of the 16S rRNA gene, has provided increasingly robust evidence for the existence of a non-diseased HBM (Dinakaran et al., 2014; Damgaard et al., 2015; Gosiewski et al., 2016; Païssé et al., 2016; Kowarsky et al., 2017; Whittle et al., 2018; Qiu et al., 2019). RNA-sequence data have also contributed to this premise, as bacterial transcripts have been identified in non-diseased control groups (Loohuis et al., 2018; Whittle et al., 2018). Researchers characterizing the blood-microbiome in diseased patients, largely through culture-independent methods, have also detected genetic material in their healthy control groups (Table 1). Moreover, the presence of comparable bacterial phyla in different studies appears to lend support for the existence of a healthy HBM (McLaughlin et al., 2002; Amar et al., 2011, 2013; Dinakaran et al., 2014; Païssé et al., 2016; Li et al., 2018; Loohuis et al., 2018; Whittle et al., 2018; Qiu et al., 2019).

In addition to challenging the *status quo* of the “germ-free” human blood paradigm, methodological obstacles have hindered HBM research. Many micro-organisms found naturally within human blood may in fact be in a dormant state (Potgieter et al., 2015). Accordingly, culture-based methods cannot be reliably employed to support the existence of a HBM. Furthermore, while the concentration of bacterial DNA in the blood is typically very low, increasingly-sensitive analytical techniques, particularly qPCR, and targeted NGS may substantiate current evidence for the presence of “innocuous” bacterial taxa in the blood of healthy humans (Païssé et al., 2016).

However, rigorous experimental controls, which are essential when studying low-biomass microbiomes prone to contamination from external sources, are not routinely included. This is particularly problematic as the detection of >90 microbial genera in DNA extraction and library preparation controls (Salter et al., 2014; Lauder et al., 2016) highlights the influence that contaminants derived from reagents and laboratory environments exert on sequence-based HBM analyses. The analyses of negative DNA extraction controls, as performed by Moriyama et al. (2008), showed significantly less 16S rRNA gene amplification when compared to blood derived from healthy individuals. These controls comprised saline water which had been in contact with povidone iodine sterilized skin (Moriyama et al., 2008). In another study by Dinakaran et al. (2014), the analyses of comparable samples as negative controls for 16S rRNA gene targeted Illumina MiSeq WMGS showed virtually no amplified “contaminant” taxa. Although a number of these samples did yield >10,000 DNA sequence reads, their taxonomic composition differed significantly from that of both the diseased and healthy blood samples (Dinakaran et al., 2014). While investigating the blood-microbiome of cirrhotic patients, Traykova et al. (2017) recovered bacterial DNA from the blood of >20% of the healthy cohort. In this study, sterile water and pan-bacterial assays, which detect a broad range of bacterial taxa, were used as negative and positive controls. The use of controls was also implemented when the blood-microbiome in different blood fractions was characterized (Païssé et al., 2016). In a recent study by Loohuis et al. (2018) the importance of including stringent controls when studying low biomass microbiomes, such as human blood, was clearly demonstrated. While investigating the blood-microbial transcriptomes of both healthy human individuals and of patients affected with brain disorders, RNA obtained from lymphoblast cell lines were used as negative controls, and cells infected with *Chlamydia*, as positive controls. RNA reads were identified only for the *Chlamydiae* phylum in the positive controls, and no microbial sequences were detected in the lymphoblast cells.

It is evident that further research is required to establish whether the microbial DNA and RNA found in healthy human blood represents either living or dead, or active or non-active bacterial taxa. Although contamination derived from human sources poses a significant challenge to blood-microbiome research, bacteriological activity in the blood could potentially be studied through viability assay techniques such as propidium monoazide (PMA) treatment and cellular energy measurements (Emerson et al., 2017). However, there is presently no specific and reliable means of detecting living bacteria in human blood.

Despite the fact that evidence for the presence of bacterial taxa comprising a healthy blood-microbiome in humans is accumulating, not much is known about the presence of other micro-organisms, such as viruses, Archaea and lower eukaryotes (i.e., fungi) in the blood of healthy humans. The presence of archaeal DNA is generally not reported (Nikkari et al., 2001; McLaughlin et al., 2002; Moriyama et al., 2008; Dinakaran et al., 2014; Damgaard et al., 2015; Gosiewski et al., 2016; Païssé et al., 2016), presumably due to low abundance or the complete absence of archaea from blood samples. Dinakaran et al. (2014) did, however, document a relative abundance of 0.01% circulating archaeal DNA in the blood plasma of healthy individuals. The fungal microbiomes of the human intestinal tract, mouth, skin, lungs, and other body-sites have been explored (Cui et al., 2013; Huffnagle and Noverr, 2013). As the presence of fungi in the blood of healthy individuals has only recently been reported (Panaiotov et al., 2018), more research concerning the human blood myco-biome, in particular studies including stringent negative controls, is required. Regarding a tentative human blood-virome, and following the exclusion of taxa attributable to contamination, Moustafa et al. (2017) recovered 19 viral taxa from 42% of overtly non-diseased individuals. Previous reports have confirmed the presence of eukaryotic viruses, such as rhabdoviruses (Stremlau et al., 2015), anelloviruses (Furuta et al., 2015), and other families including *Herpesviridae* and *Poxiviridae* (Rascovan et al., 2016) in healthy human blood. Additional research concerning the human blood-virome is therefore required to determine whether viruses are resident members of the HBM, or simply remnants of previous infections.

## Origin and Location of Blood-Borne Bacteria

It is not known whether blood-borne bacteria are exploiting a viable ecological niche, or whether they are simply transient residents in the blood. Some researchers suggest that the presence of bacteria in blood is a consequence of translocation from other body-sites, particularly the gastro-intestinal tract (Païssé et al., 2016). Indeed, the etiology of diabetes, cardiovascular disease, hematological disorders and cirrhosis has been ascribed to the translocation of bacteria from the intestinal tract, primarily via the intestinal epithelial mucosa (Amar et al., 2013; Dinakaran et al., 2014; Sato et al., 2014; Manzo and Bhatt, 2015; Traykova et al., 2017). Correspondingly, it has been suggested that bacteria derived from the skin- (Cogen et al., 2008) and oral-microbiomes could also diffuse into the blood when the barriers between these environments and the circulatory system are compromised (Forner et al., 2006; Bahrani-Mougeot et al., 2008; Iwai, 2009). Recently, Whittle et al. (2018) compared microbial DNA data derived from healthy individuals to HMP microbiome data. They demonstrated that, whereas the blood-microbiome closely resembles the skin- and oral microbiomes, it differs substantially from the intestinal microbiome. While most studies tend to consider the diffusion of bacteria into the blood-circulatory system as exceptional, this phenomenon may therefore occur rather frequently in healthy individuals, supporting the recent findings of a healthy HBM (Moriyama et al., 2008; Païssé et al., 2016). Even if the intestinal epithelial membrane is not compromised, other mechanisms could facilitate the entry of intestinal bacteria into the circulatory system. Micro-organisms can be absorbed by dendritic cells (antigen or “accessory” cells of the mammalian immune system) and transported through the intestinal epithelium (Rescigno et al., 2001; Niess et al., 2005) or via the assistance of intestinal mucus-secreting goblet cells (McDole et al., 2012). Additionally, intestinal M-cells (specialized epithelial cells of the mucosa-associated lymphoid tissues) could also be involved in the translocation of bacteria from the intestinal lumen to the blood-circulatory system (Vazquez-Torres et al., 1999; Jang et al., 2004; Lelouard et al., 2012).

The vertical transmission of the microbiome is an almost universal phenomenon in the animal kingdom (Funkhouser and Bordenstein, 2013) and, as for the human IM, blood-borne bacteria could also have a maternal origin. Although fetal and maternal blood does not mix during gestation, bacteria could colonize the fetal circulatory system even before delivery. Following the isolation of bacterial DNA from the umbilical cords of healthy new-borns delivered by C-section, Jiménez et al. (2005), suggested the tentative existence of a pre-natal blood-microbiome. Blood from 20 neonates was collected and processed in a class II safety cabinet to avoid contamination. Thereafter, 16S rRNA gene sequencing was performed from colonies obtained after culturing in brain and heart infusion broth (Jiménez et al., 2005). Appropriate negative controls were included in the culturing and sequencing steps. The presence of micro-organisms in the blood of new-born humans could be derived from other body-sites, *in utero*, such as fetal intestinal or oral sites. Thus, while it is widely believed that the initial new-born inoculum stems from contact with the vaginal-, fecal- or skin-microbiota of the mother during labor and that this infant-microbiome is subsequently enriched through breastfeeding (Penders et al., 2006; Biasucci et al., 2008; Dominguez-Bello et al., 2010; Azad et al., 2013), some evidence indicates the existence of a fetal microbiome *in utero* and the enrichment of that original set of micro-organisms following birth (Romano-Keeler and Weitkamp, 2014). Although controversial, various research groups have suggested the presence of bacteria in the placenta (Aagaard, 2014; Aagaard et al., 2014), the amnion (Hitti et al., 1997; Bearfield et al., 2002; DiGiulio et al., 2010), fetal membranes (Steel et al., 2005) and the meconium (Jiménez et al., 2008; Gosalbes et al., 2013; Moles et al., 2013), favoring this hypothesis (Funkhouser and Bordenstein, 2013). While the mechanisms implicated in the subsequent transmission of bacteria from other body-sites to the fetus are unknown, one possible route of entry into the fetus could involve the ingestion of amniotic fluid during gestation (Romano-Keeler and Weitkamp, 2014). The possible maternal origin of the blood-microbiome requires further investigation.

It is evident that the exact origin of the HBM remains to be fully elucidated. It could, hypothetically, derive from the mother preceding birth, or from the translocation of micro-organisms derived from other sources after birth and during the normal human lifecycle. As with the human IM, and besides the influence on IM taxonomic composition of diet (De Filippo et al., 2010), age (Yatsunenko et al., 2012), seasonal variation (Smits et al., 2017), and host immuno-modulation (Thorburn et al., 2014), the HBM may well comprise an adaptive micro-ecological system prone to environmental influences and exposure to novel microbial taxa. Accordingly, if micro-organisms present in human blood do originate from other body-sites, and if bacterial translocation is not an infrequent event, it appears reasonable to assume that the healthy HBM is highly dynamic. Be that as it may, recent findings based predominantly on cross-sectional studies targeting the 16S rRNA gene show a set of dominant blood-borne bacterial phyla (i.e., *Proteobacteria*, followed by *Actinobacteria, Firmicutes*, and *Bacteroidetes*) (McLaughlin et al., 2002; Amar et al., 2011, 2013; Dinakaran et al., 2014; Gosiewski et al., 2016; Païssé et al., 2016; Loohuis et al., 2018; Whittle et al., 2018; Qiu et al., 2019) and are indicative of longer-term HBM stability.

With regards the precise location of micro-organisms inside human blood, current evidence suggest that bacterial taxa can survive inside both erythrocytes and leukocytes. *Chlamydia pneumoniae*, an intracellular bacterium and the major causative agent of pneumonia, has been found to inhabit peripheral blood mononuclear cells (PBMCs) in healthy individuals (Yamaguchi et al., 2004). Other bacteria, for example *Staphylococcus aureus*, can also invade and persist in white blood cells (WBCs). As far back as 2000, Gresham et al. (2000) showed that these bacteria both reside and retain their virulence within neutrophils. Thwaites and Gant (2011) have also suggested that WBCs, and especially neutrophils, could act as “Trojan horses” by offering protection against human antibodies, thereby facilitating the dissemination of *S. aureus* to different body-sites. Moreover, when Païssé et al. (2016) analyzed the blood-microbiome of healthy individuals, most bacterial DNA (93.74%) was found to be localized within the buffy coat (BC), which consists primarily of WBCs and platelets. A correlation between leukocyte concentration and the number of 16S rRNA gene copies in the BC of study participants was also identified. Similarly, some bacteria can enter RBCs directly, and persist within the nutrient-rich environment; it has been shown that *Staphylococcus aureus*, a species commonly found in both healthy and diseased human IM (Grice et al., 2009), can utilize iron (Fe) present in RBCs as a nutrient source (Yamaguchi et al., 2013). Yamaguchi et al. (2013) also showed that *Streptococcus pneumoniae*, a bacterium implicated in the onset of pneumonia and sepsis, became increasingly viable when incubated with erythrocytes. Similarly, it has been reported that *Brucella melitensis*, the causative agent of ovine brucellosis, and *Francisella tularensis*, a Gram-negative bacterium that causes tularaemia, also possess the capacity to invade and persist in erythrocytes (Horzempa et al., 2011; Vitry et al., 2014).

## Composition of the Putative Healthy Human Blood-Microbiome

Despite the fact that the existence of a blood-microbiome in healthy human individuals appears to be supported by recent studies, knowledge of the phylogenetic diversity of blood-borne bacteria remains limited. In contrast to the dominant bacterial phyla typically observed in the human IM (i.e., *Firmicutes* and *Bacteroidetes*), the HBM appears to be dominated by the phyla *Proteobacteria* followed by *Actinobacteria, Firmicutes*, and *Bacteroidetes* (McLaughlin et al., 2002; Amar et al., 2011, 2013; Dinakaran et al., 2014; Gosiewski et al., 2016; Païssé et al., 2016; Li et al., 2018; Loohuis et al., 2018; Whittle et al., 2018; Qiu et al., 2019). The characterization of blood bacterial diversity, however, varies between studies. In 2008, Moriyama et al. (2008) identified a set of bacterial taxa in their study of bacteria from the blood of two healthy individuals comprising mostly *Bacillus, Flavobacteria, Stenotrophomas* and *Serratia*. Using a culture-based approach, Damgaard et al. (2015) observed bacterial growth in the blood of ~62% of healthy individuals. The most prominent taxa detected were *Propionibacterium acnes* and *Staphylococcus epidermis*, as well as *Bacilli* and *Micrococcus* species (Damgaard et al., 2015). In 2016, Païssé et al. (2016) analyzed bacterial DNA present in different fractions of human blood. At class level, *Fusobacteria* and *Flavobacteria* were more abundant in RBCs, while members of the *Clostridia* class were dominant in plasma and erythrocyte fractions. Seven genera were identified in the RBC fraction, including two opportunistic pathogens, namely *Acinetobacter baumanni* and *Stenotrophomonas maltophilia*. Problematically, although the incidence of diverse blood taxonomic composition might indeed reflect actual microbial configuration, bacterial DNA found to contaminate DNA extraction kits typically includes *Bacillus, Flavobacteria, Fusobacteria, Propionibacterium*, and *Serratia* (Glassing et al., 2016). In addition to a critical awareness of potentially contaminating taxa, there is also a need for much broader metagenomic studies encompassing larger cohorts of both healthy and diseased individuals, as these would provide valuable insights into the composition of the putatively “healthy” HBM as well as its functionality and potential role in maintaining optimal human health and the onset of disease.

## The Clinical Relevance of the Healthy HBM

With reference to the role of the human microbiome in pathogenesis, the concept of “dysbiosis,” which refers to a change in the composition of symbiotic or commensal microbial communities (Petersen and Round, 2014), is particularly relevant. Although it is not known whether dysbiosis is a cause, or simply a reflection, of a diseased state (Bäckhed et al., 2012), numerous studies have related changes in human microbial community composition with the onset of disease. Examples include diabetes (Qin et al., 2012), asthma (Teo et al., 2015), inflammatory bowel disease (Morgan et al., 2012), autism (Parracho et al., 2005) and even complex disorders such as Alzheimer's disease (Pistollato et al., 2016). While considerable research has been dedicated to address the relationship between the IM and human health, a limited number of studies have explored dysbiosis of the HBM and its potential role in pathogenesis. Conditions such as diabetes, pancreatitis and also cardiovascular- and liver-disease have, however, been related to changes in the HBM. Using an IM microbial qPCR microarray, the screening for bacterial DNA in the blood of both cirrhotic and healthy individuals by Traykova et al. (2017) resulted in the detection of higher levels of bacterial diversity in patients with cirrhosis, as opposed to a healthy control cohort. They also reported an increase of total bacterial DNA concentration in the blood of the diseased cohort compared to healthy controls. The HBM in patients with severe acute pancreatitis has also been analyzed by 16S rRNA gene amplicon sequencing (Li et al., 2018). Microbial taxonomic diversity was found to be reduced when compared to the healthy human cohort, and an increase in *Bacteroidetes* and a decrease in *Actinobacteria* were observed in pancreatitis patients. At class level, *Bacteroidia* and *Clostridia* increased in abundance, while *Actinobacteri*a, *Flavobacteria*, and *Bacilli* were reduced in the diseased group when compared to healthy controls (Li et al., 2018). These variations in dominant taxa are strongly suggestive of blood-microbiome dysbiosis in pancreatitis patients. The onset of cardiovascular diseases may also be linked to HBM dysbiosis. In 2013, Amar et al. (2013) discovered that the blood of patients who presented an acute cardiovascular event, even years following sample collection, had a significant decrease of total bacterial DNA when compared to a healthy cohort, as well as an increase in taxa assigned to the *Proteobacteria*. Accordingly, it was concluded that dysbiosis in the HBM could serve as a “marker” for CVD prediction. One year later, Dinakaran et al. (2014) also proposed the likelihood of an increase in microbial diversity and bacterial DNA concentration when analyzing cell-free DNA circulating in the blood of CVD patients. In this study, it was observed that *Actinobacteria* were dominant over *Proteobacteria* in CVD patients, while an opposite trend was observed in the healthy cohort.

The association between HBM-dysbiosis and the onset of liver-disease has also been explored (Lelouvier et al., 2016; Schierwagen et al., 2018), resulting in the proposal that blood-microbiota could serve as biomarkers for non-alcoholic fatty liver disease (NAFLD) prediction in obese patients (Lelouvier et al., 2016). In this study, qPCR and 16S rRNA gene targeted NGS was employed. Liver fibrosis patients exhibited higher concentrations of 16S rRNA gene in their blood when compared to non-diseased participants. In addition, unique bacterial taxonomic clustering was observed in patients suffering from severe liver fibrosis (Lelouvier et al., 2016). In 2018, Schierwagen et al. (2018) performed 16S rRNA gene NGS analyses on blood samples obtained from the portal vein, central and peripheral venous blood and liver outflow in patients suffering from liver fibrosis. Their findings corroborated those in the NAFLD study (Lelouvier et al., 2016). Furthermore, each of these circulatory compartments exhibited a unique taxonomic composition at genus level (Schierwagen et al., 2018).

In addition to these examples, various other studies have established possible associations between blood-derived bacteria originating from the IM, and the onset of diabetes; whereas IM bacteria have been detected to occur in ~28% of diabetes patients, healthy participants exhibited only ~4% of IM-derived bacterial taxa (Sato et al., 2014). The most abundant taxa identified in the diabetes group included *Clostridium coccoides* and the *Atopobium* cluster (Sato et al., 2014). Although Amar et al. (2011) could not convincingly demonstrate a significantly different HBM in patients prone to the development of diabetes, they did observe a higher 16S rRNA gene concentration in the blood of participants. Consequently, high concentrations of blood-derived bacterial DNA could potentially be used as a predictive marker for this condition. Recently, Qiu et al. (2019) did not find a significant difference in terms of blood bacteria diversity between type two diabetes mellitus patients and healthy individuals Nonetheless, participants containing the genus *Sediminibacterium* in their blood showed a higher risk to develop diabetes, while individuals that carried the genus *Bacteroides* had a decreased risk of presenting the disease (Qiu et al., 2019).

## Concluding Remarks and Future Prospects

The existence of a healthy HBM remains to be challenged, particularly in light of recent criticisms of, for example, the existence of a discrete human placental-microbiome (Lauder et al., 2016). A number of studies on the HBM are furthermore mired by essential shortcomings (Table 1), which casts some doubt over the validity of their results. Moreover, current definitions of “healthy” are vague and consequently problematic in terms of defining a “healthy” HBM. As with the human IM, “healthy” could be defined in terms of ecological stability (the ability to resist community change or rapidly return to a baseline state following stress-related change), an idealized (presumably “health-associated”) composition or by a most desirable functional profile (including metabolic and trophic provisions to the host) (Bäckhed et al., 2012).

Evidence indicative of the presence of a microbial component in the blood of healthy human individuals is, nevertheless, steadily accumulating and recent studies have identified comparable bacterial phyla in the blood of healthy individuals (McLaughlin et al., 2002; Amar et al., 2011, 2013; Dinakaran et al., 2014; Gosiewski et al., 2016; Païssé et al., 2016; Li et al., 2018; Loohuis et al., 2018; Whittle et al., 2018; Qiu et al., 2019). Not discounting the shortcomings of each of the cited studies, a coherent picture of the healthy HBM emerges when one considers the positive aspects introduced by each study. For example, the inclusion of microscopy (McLaughlin et al., 2002) and the application of both DNA (Dinakaran et al., 2014; Païssé et al., 2016; Li et al., 2018; Whittle et al., 2018) and RNA analyses to the blood of healthy individuals (Loohuis et al., 2018; Whittle et al., 2018). From the literature reviewed here, and taking into consideration the trend toward including analytical (positive and negative) controls in HBM-related studies, we conclude that the notion that a healthy (non-diseased) human blood-microbiome exists cannot simply be discarded.

With regards to future studies on the “healthy” HBM, we recommend thorough experimental designs that ensure the reduction of both contamination and technical biases. For this, researchers must improve the protocols implicated in obtaining (drawing blood), processing (using kits and storage), and generating (sequencing protocols and techniques) both healthy and diseased blood microbial data. This could be achieved by studying the blood microbiome together with that of the skin around the puncture site from where the blood was obtained, as well as characterizing microbial DNA derived from potential microorganisms present in the needle, vacutainers, reagents, and other consumable items used during sampling.

We furthermore encourage researchers to investigate this unique microbiome, as it promises to hold the potential to facilitate both the diagnosis and improved understanding of the onset of numerous human diseases. It is still unclear whether the putative healthy HBM comprises a core set of bacterial taxa, or a dynamic and adaptive group of micro-organisms. We recommend performing studies that also take into consideration the “time” element in the HBM. Most of the literature reviewed here deals with snapshots of blood bacterial communities, potentially overlooking important changes of the HBM across time. In addition, the influence of age, geography and socio-economic status (e.g., access to nutritional diet and healthcare services) on healthy HBM composition remains ambiguous. With reference to the location of micro-organisms in the human blood-circulatory system, it is conceivable that bacteria either reside inside, or adhere to blood cells or in the plasma fraction of the blood. Moreover, with regards to the origin of the healthy HBM, it appears that blood-borne micro-organisms, particularly bacteria, may well originate from various body-sites and that many might have a maternal (*i.e*., vertical) origin. Samples from the skin-, oral- and Intestinal-microbiomes should be analyzed at the same time as blood samples to gain insight into the potential origin of these microbes. Finally, in order to ascertain the potential roles and functions linked to bacteria and other microorganisms in the human blood, studies on the HBM based on WMGS are undoubtedly essential. The increasing recognition of the existence of a healthy HBM stimulates novel and diverse research avenues, some of which may well turn out to be of considerable clinical significance.

## Author Contributions

DC initiated and conceived the review, investigated the literature, and wrote the review. RR contributed to the writing and critical revision of the manuscript. DAC contributed to revision of the manuscript. MP directed and contributed to the review design, investigated the literature, and contributed to the critical revision of the manuscript.

### Conflict of Interest Statement

The authors declare that the research was conducted in the absence of any commercial or financial relationships that could be construed as a potential conflict of interest.
